# Payers' Views of the Changes Arising through the Possible Adoption of Adaptive Pathways

**DOI:** 10.3389/fphar.2016.00305

**Published:** 2016-09-28

**Authors:** Michael Ermisch, Anna Bucsics, Patricia Vella Bonanno, Francis Arickx, Alexander Bybau, Tomasz Bochenek, Marc van de Casteele, Eduardo Diogene, Jurij Fürst, Kristina Garuolienė, Martin van der Graaff, Jolanta Gulbinovič, Alan Haycox, Jan Jones, Roberta Joppi, Ott Laius, Irene Langner, Antony P. Martin, Vanda Markovic-Pekovic, Laura McCullagh, Einar Magnusson, Ellen Nilsen, Gisbert Selke, Catherine Sermet, Steven Simoens, Robert Sauermann, Ad Schuurman, Ricardo Ramos, Vera Vlahovic-Palcevski, Corinne Zara, Brian Godman

**Affiliations:** ^1^Pharmaceuticals Department, National Association of Statutory Health Insurance FundsBerlin, Germany; ^2^Department of Finance, University of ViennaVienna, Austria; ^3^Independent Pharmaceutical ConsultantMellieha, Malta; ^4^National Institute for Health and Disability InsuranceBruxelles, Belgium; ^5^Zilveren Kruis AchmeaLeiden, Netherlands; ^6^Department of Drug Management, Faculty of Health Sciences, Jagiellonian University Medical CollegeKrakow, Poland; ^7^Clinical Pharmacology Service, Vall d'Hebron University Hospital, Fundació Institut Català de FarmacologiaBarcelona, Spain; ^8^Medicinal Products Department, Health Insurance Institute of SloveniaLjubljana, Slovenia; ^9^Department of Pathology, Forensic Medicine and Pharmacology, Faculty of Medicine, Vilnius UniversityVilnius, Lithuania; ^10^Medicines Reimbursement Department, National Health Insurance FundVilnius, Lithuania; ^11^National Health Care InstituteDiemen, Netherlands; ^12^State Medicines Control AgencyVilnius, Lithuania; ^13^Health Economics Centre, University of Liverpool Management SchoolLiverpool, UK; ^14^Scottish Medicines ConsortiumGlasgow, UK; ^15^Pharmaceutical Drug Department, Azienda Sanitaria Locale of VeronaVerona, Italy; ^16^State Agency of MedicinesTartu, Estonia; ^17^Wissenschaftliches Institut der AOK (WIdO)Berlin, Germany; ^18^Ministry of Health and Social WelfareBanja Luka, Bosnia and Herzegovina; ^19^Department of Social Pharmacy, Medical Faculty, University Banja LukaBanja Luka, Bosnia and Herzegovina; ^20^Pharmacoeconomics and Health Technology Assessment, Department of Pharmacology and Therapeutics, Trinity CollegeDublin, Ireland; ^21^Department of Health Services, Ministry of HealthReykjavík, Iceland; ^22^Norweigian Directorate for HealthOslo, Norway; ^23^Institut de Recherche et Documentation en Economie de la Santé (IRDES)Paris, France; ^24^Department of Pharmaceutical and Pharmacological Sciences, KU LeuvenLeuven, Belgium; ^25^Department of Pharmaceutical Affairs, Main Association of Austrian Social Insurance InstitutionsVienna, Austria; ^26^Health Technology Assessment, Prices and Reimbursement Department, INFARMED - National Authority of Medicines and Health Products, I. P., Parque de Saúde de LisboaLisbon, Portugal; ^27^Department of Clinical Pharmacology, University HospitalRijeka, Croatia; ^28^Barcelona Health Region, Catalan Health ServiceBarcelona, Spain; ^29^Department of Pharmacoepidemiology, Strathclyde Institute of Pharmacy and Biomedical Sciences, University of StrathclydeGlasgow, UK; ^30^Division of Clinical Pharmacology, Department of Laboratory Medicine, Karolinska Institutet, Karolinska University Hospital HuddingeStockholm, Sweden

**Keywords:** EMA, adaptive pathways, payers, marketing authorization, Europe

## Abstract

Payers are a major stakeholder in any considerations and initiatives concerning adaptive licensing of new medicinal products, also referred to as Medicines Adaptive Pathways to patients (MAPPs). Firstly, the scope and necessity of MAPPs need further scrutiny, especially with regard to the definition of unmet need. Conditional approval pathways already exist for new medicines for seriously debilitating or life-threatening diseases and only a limited number of new medicines are innovative. Secondly, MAPPs will result in new medicines on the market with limited evidence about their effectiveness and safety. Additional data are to be collected after approval. Consequently, adaptive pathways may increase the risk of exposing patients to ineffective or unsafe medicines. We have already seen medicines approved conventionally that subsequently proved ineffective or unsafe amongst a wider, more co-morbid population as well as medicines that could have been considered for approval under MAPPs but subsequently proved ineffective or unsafe in Phase III trials and were never licensed. Thirdly, MAPPs also put high demands on payers. Routine collection of patient level data is difficult with high transaction costs. It is not clear who will fund these. Other challenges for payers include shifts in the risk governance framework, implications for evaluation and HTA, increased complexity of setting prices, difficulty with ensuring equity in the allocation of resources, definition of responsibility and liability and implementation of stratified use. Exit strategies also need to be agreed in advance, including price reductions, rebates, or reimbursement withdrawals when price premiums are not justified. These issues and concerns will be discussed in detail including potential ways forward.

## Introduction

In most European countries, healthcare is seen as a public good, inasmuch as universal healthcare is a stated or accomplished goal—be it through a government-funded system or mandatory health insurance^1^. Pharmaceuticals are a major component of healthcare, and their provision increasingly challenges healthcare systems^2^ (Malmstrom et al., 2013). Consequently, it is no surprise that those responsible for payment or reimbursement of medicines, and their advisors (collectively referred to as “payers”), carefully observe developments at the European level including initiatives of the European Medicines Agency (EMA). Since the provision of healthcare is subject to the principle of solidarity and therefore a national issue, any projects involving access to medicines from the moment of marketing authorization, especially for new premium priced medicines, are subject to particular scrutiny from their perspective (Godman et al., 2015; Matusewicz et al., 2015).

EMA started a pilot on adaptive licensing, later renamed to adaptive pathways, in March 2014. The rationale for MAPPs, and the project itself, have been described in scientific publications under the participation of EMA scientific officers as well as on EMA's website (Eichler et al., 2015)^3^. Briefly, adaptive pathways should be a holistic approach to medicines' approval, based on the premise that approval can be done iteratively, i.e., a medicine can at first be either approved for a small group of patients, if there is enough compelling evidence, and later approved for a larger patient group based on the evidence collected after the approval for the initial group. Alternatively, a medicine could be approved on the basis of preliminary clinical data, such as surrogate endpoints, e.g., biomarkers or response rates, which would ultimately need to be verified with the help of clinical data reflecting actual patient benefit such as increased length of survival in patients with advanced cancer or a reduction in cardiac events (Davis et al., 2016). Adaptive pathways are not meant to be a new route of approval for medicines, but to make use of existing approval tools, in particular conditional marketing authorization^4^.

To support MAPPs, IMI (the Innovative Medicines Initiative) initiated ADAPT SMART as an enabling and coordination platform for MAPPs^5^^,^^6^. IMI is a joint initiative between the European Union and the European Pharmaceutical Industry Association—EFPIA^5^. However, the MAPPs approach is evolving and it is currently controversial. We not only see criticism from independent scientists and organizations^7^ (Davis et al., 2016; Hawkes, 2016a,b; EPHA^8^; IQWiG^9^), but also publications from the EMA sphere contradicting official EMA statements (Eichler et al., 2012; 2015).

In August 2016, the EMA published a final report on the Adaptive Pathways pilot^4^. All discussions within this pilot were confidential so neither the precise contents of these discussions, nor all the companies involved, are publicly known. The report describes the experience of selecting seven appropriate projects from 62 initial applications and of six multi-stakeholder consultations on the development pathway. It emphasizes the feasibility and usefulness of jointly agreeing on a data generation plan which meets the needs of regulators and health technology assessors. However, the report acknowledges the limitations of MAPPs - key unsolved issues of “real-life” data generation, prescriptions management, managed entry agreements (MEAs), and how to leave the adaptive pathway if this becomes necessary. The report also highlights the need to further address the involvement of patients, healthcare professionals and payers.

These developments and suggestions are in addition to the PRIME project, another new initiative from the EMA, to foster research on and development of medicines that have the potential to address an unmet medical need^10^.

Unlike other goods, medicinal products need to obtain a regulatory approval (marketing authorization) before being placed on the market. This is justified by the fact that medicinal products inevitably carry risks for patients and the general population. Consequently, a special need for public protection through a risk/benefit assessment is universally acknowledged. The current marketing authorization procedure emerged as a response to a number of disasters such as sulfanilamide or thalidomide (Contergan®)^7^, (Banzi et al., 2015). The European regulatory system has been built to protect public health and to preserve the confidence of patients and the medical professions that, properly prescribed, the benefit of every authorized medicine outweighs its risks.

The introduction of MAPPs is being undertaken under the current legal framework of the Community system for authorization of medicinal products [Commission Regulation (EC) No 726/2004 and Commission Regulation (EC) No 507/2006]. Procedures other than the standard authorization procedures such as conditional approval (Banzi et al., 2015) should offer the same risk governance and level of public health protection as standard procedures. Lowering the standards for risk governance and marketing authorization could be in breach of the Treaties and put patients at risk, especially given the many recent examples where observational studies have suggested a treatment benefit only to be overturned by RCTs (Davis et al., 2016).

This paper explains the concerns of payers regarding the MAPPs initiative. These build on the concerns of others (ISDB, 2016) and revolve around the scope of the initiative, its execution, and its consequences for payers—and, ultimately, European society, meaning patients and those who contribute to the financing of healthcare. It also discusses possible ways forward.

## Areas of concern

### Scope and rationale for MAPPs—justification of the concept for adaptive pathways

The EMA final report on adaptive pathways pilot describes MAPPs as an iterative development plan that will initially target a well-defined group of patients that is likely to benefit most from the new treatment (Davis et al., 2016, EMA^4^). This is supposed to be followed by iterative phases of evidence gathering and progressive licensing adaptations, concerning both the authorized indication and the potential further therapeutic uses of the medicine, to expand its use to a wider patient population as more data becomes available. Our understanding is that this means initial marketing authorization is to be granted for promising populations based on phase II (or earlier) clinical data. It implies granting marketing authorization and, ultimately, reimbursement, based principally on surrogate endpoints. We are aware that whilst phase II trials do not always measure surrogate endpoints, any clinical endpoint data may be immature. We understand this to mean that speeding up access to new medicines via adaptive pathways is to be achieved by putting aside traditional phase III clinical trials in favor of post-marketing evidence generation. This requirement is problematic since only a very limited subset of surrogate endpoints have been validated to date and relying on unvalidated surrogate endpoints seriously increases uncertainty and is generally advised against (EPHA^8^, Mangiapane and Velasco Garrido, 2009; Velasco Garrido and Mangiapane, 2009; Svensson et al., 2013; Henshall et al., 2014; Naci et al., 2015; Prasad et al., 2015; IQWiG^11^). Examples of concerns include extrapolating surrogate markers in patients with advanced solid tumors such as disease free survival to overall survival (Tuma, 2009; Kantarjian et al., 2013; Cortazar et al., 2014; Prasad et al., 2015).

### Equity and allocation of public resources in the light of increased uncertainty

Accepting such clinical and economic uncertainties can only be justified for new medicines meant for patients with high unmet medical need, especially those with rare diseases and if patient-relevant outcomes cannot be explored within a reasonable amount of time. However, “unmet medical need” is currently not precisely defined, although attempts have been made when assessing priority areas for new medicines (Kaplan et al., 2013; Banzi et al., 2015). For regulators and payers to be aligned, there needs to be a common agreed position for unmet medical need. This definition should be based upon a public health perspective and provide a balanced, level playing field, and the same level of risk governance for different disease conditions.

MAPPs suggest an initial marketing authorization for a restricted patient population upon demonstration of a positive benefit/risk balance. However, one has to consider that even for seriously ill patients, being treated with a new medicine with poorly characterised benefits, and which might only cause harm, should not be part of everyday care. Routine treatment, unlike testing in clinical trials, should be guided by known outcomes and not by limited or lack of knowledge.

The extent of uncertainty which patients would have to accept if new medicines were to be authorised earlier is illustrated by available data. The combined success rate at phase III and submissions is only approximately 50%. Two thirds of terminations of new medicines are due to lack of efficacy, more than 20% due to safety issues (Arrowsmith, 2011). This suggests that preliminary data from earlier phases of development typically overestimate the potential benefits of a new treatment, and do not allow a robust assessment of risks and benefits. There are also a number of examples of new medicines that were given marketing authorization on the basis of the regular data package but were proven to be ineffective or unsafe when used in routine clinical care (Eichler et al., 2011; Godman et al., 2015). The latter examples have been used to demonstrate the possibility of foregoing some of the current requirements for marketing authorization in exchange for better investigation after approval in a wider more co-morbid population likely to receive the new medicine. However, in view of the failure rates in phase III trials, including withdrawals due to safety concerns, this scenario is incomplete. If promising data after phase II were sufficient for marketing authorization, a larger number of ineffective or unsafe medicines - often at higher prices than current standards - would have been approved. To our knowledge, no adequate measures to safeguard against this have been proposed for MAPPs.

According to the EMA report on MAPPs, adaptive pathways seek to balance timely access for patients who are likely to benefit most from the new medicine with the need to provide adequate evolving information on the benefits and risks of the new medicine itself^4^. Publications arising from the EMA suggest that the EMA is of the opinion that earlier access to new medicines is necessary and that adaptive pathways are a key way to achieve this goal. We have concerns with this belief. This is because the number of new medicines that have been approved by EMA has been quite stable in recent years with no discernible decline indicating problems in the approval process^12^. Consequently, we see little need to change this. Pharmaceutical companies should as a matter of course already be seeking ways to optimize development cycles. Lastly, the potential to speed up market access for new medicines when there is true unmet medical need already exists. This includes compassionate use programs or named patient programs^7^ (Baird et al., 2014). Consequently, there should be objective and scientifically valid justifications for new measures such as adaptive pathways to ensure protection of patients^8^ (Davis et al., 2016; Hawkes, 2016a). As a result, the current rationale for MAPPs does not appear compelling.

### The risk-governance framework—how beneficial are MAPPs?

Earlier approval, which is already in existence, has for instance increased the number of black-box warnings and withdrawals in the US7 (Frank et al., 2014). In addition Prescrire, an independent drug information journal, recently assessed 22 medicines “approved conditionally” in the EU since 2006. They believed 27% were “not acceptable,” such as medicines without evident benefit but with potential or real disadvantages, 28% as having “judgment reserved,” i.e., assessments reserved until better data becomes available, 9% as “nothing new,” 18% as “possibly helpful” and only 18% as “offering an advantage”^13^. There have also been concerns with five out of the six orphan medicines receiving marketing authorisation in 2004 (Joppi et al., 2016).

Existing ways to speed up approval for marketing authorization (like conditional or exceptional approval) already pose problems for conducting health technology assessments (HTAs) and implementing measures such as valuing new medicines based on the data presented. Earlier access means inherently less available data at the time of approval. This lack of information impedes assessments of the therapeutic value of new medicines and thereby decisions on value-based pricing, exacerbated by concerns with defining terms such as innovation (Annemans et al., 2012). This has important implications for ever-increasing requested prices, especially new medicines for cancer and for orphan diseases (Cohen and Felix, 2014; Kelly and Smith, 2014; Godman et al., 2015; Howard et al., 2015). While up to now marketing authorization decisions and information are generally used as the baseline for reimbursement decisions, this position will need to be reconsidered in the framework for HTA for new medicines subject to the MAPPs process. This will create considerable challenges in already resource constrained healthcare systems.

EMA noted that adaptive licensing is already happening within the current framework as part of conditional approvals or initial approval as last-line treatment with clinical trials for earlier stages of disease to be completed at a later point in time^7^ (Banzi et al., 2015; Cerreta, 2015). Currently, these approaches are limited to special cases such as life-threatening or seriously debilitating diseases^3^. However, there are major concerns among payers if adaptive licensing becomes the preferred approach in the future (Eichler et al., 2015; Davis et al., 2016; Hawkes, 2016a,b). These concerns are heightened by the opinion of some that not only immediately life-threatening diseases have an unmet medical need, but in the end all diseases have a “treatment window of opportunity.” “The urgency of access to promising treatments is independent of disease dynamics: for every year without access, the window will shut on one annual cohort of patients, whether the window is short or long” (Eichler et al., 2015). This would open the possibility of adaptive pathways being considered for all new medicines across populations. Due to the inherent uncertainty of earlier access, greater risks to patients are expected to arise from this uncertainty. Payers and their advisers are concerned that this paradigm shift in the regulatory process could result in a breach of the legislative decision on limiting conditional approvals, confirmed in the context of the pharmacovigilance legislation in 2010. Even if MAPPs were limited to special cases, payers would need to actively consider updates to the framework to optimize the managed entry of new medicines in areas of high unmet need. This would create considerable challenges within countries. Consequently, earlier access even under the current legislative environment harbors risks and should only be considered under exceptional circumstances.

### Pitfalls of post-authorisation evidence generation—ensuring efficacy and effectiveness

Instigating MAPPs also means that more evidence is to be gathered after marketing authorization. This is of concern since it is often difficult to recruit patients for post authorization studies^7^. Additional concerns with this approach have been highlighted in a recent publication (Banzi et al., 2015). The authors demonstrated that the obligations imposed by the EMA for conditional approvals are typically fulfilled only with delay and often incompletely, if ever, by pharmaceutical companies. It has been said that ensuring the fulfillment of obligations like post-approval evidence generation is one of the core issues to be addressed in adaptive pathways, and that these obligations will be legally binding. However, if there are already delays and concerns with such schemes, it is difficult to see how this will change especially with more medicines subject to this route.

Consequently, again we believe MAPPs should only apply to areas where there is an agreement between all concerned parties (including payers) that there is a true considerable unmet medical need that may be ameliorated without creating new problems and that all the uncertainties with the new medicine have been fully communicated and concerns with existing schemes addressed (Hawkes, 2016a).

### Stratification of the use of a medicine

Stratified medicines, especially fragmentation of populations and better understanding of diseases, have been described as drivers and enablers of adaptive pathways (Eichler et al., 2015). Stratified medicine undoubtedly results in smaller treatment populations. Due to these smaller populations, RCTs are seen by some as unfeasible. We strongly dispute this in most situations given the appreciable number of patients that exist across Europe within the current definition for orphan diseases (Garattini, 2012), and pharmaceutical research and development being planned globally. On this scale, most potentially targeted populations are big enough to support randomized trials as shown in a recent study demonstrating randomisation in studies for new medicines for orphan diseases (Picavet et al., 2013). This is further supported by publications suggesting that a number of medicines for orphan diseases have now reached blockbuster status (Kesselheim et al., 2012).

Additionally, stratification promises to provide a population where the chances of increased effectiveness in terms of reducing the numbers needed to treat, or alternatively, increasing the numbers needed to harm, are improved (Godman et al., 2013). This should further improve the chances of showing clinically relevant differences in relatively small trials.

Stratified medicine combined with better understanding of diseases is supposed to open opportunities for extrapolation of results from one population to another.

In this context, basket trials of patients with different diseases harboring the same genomic aberration that is supposed to be causative to their diseases have been exemplified as modern trial designs fitting adaptive pathways (Redig and Janne, 2015). Payers are wary of this especially if companies use small populations to drive up requested prices^14^ (Godman et al., 2013). Even when for example a specific cancer mutation has been shown to be causative in some entities, response in other cancer entities harboring the same mutation was not predictable (Hyman et al., 2015). This clearly demonstrates the limits to the extent of possible extrapolations during approval.

### Limiting the use of a medicine to specific patients—prescription control

We have explained our concern that early availability of new, untested medicines, among the general population, which typically exhibits greater co-morbidities and higher age than the well-controlled population entered into phase II and III clinical trials, may jeopardize patient safety (Joppi et al., 2013; Malmstrom et al., 2013). In an environment where medicines are approved for small, well defined populations early on and evidence of their potential benefits and risks for a wider population is to be generated after this approval, restricting the use of these medicines is essential. This is why EMA's report emphasizes the need for prescription control^4^. However, it is left very unclear how this is to be accomplished.

Limiting the use of medicines to approved populations is challenging (Davis et al., 2016). Payers can limit reimbursement for use of medicines outside approved populations; however, enforcement of these restrictions can be difficult in a number of European countries without specific regulations in place (Godman et al., 2010). Any added costs and use of resources due to unwarranted use of medicines outside of approved populations will be a burden to payers especially if requested prices are higher than current standards. Several member states also currently do not provide a legal framework to limit doctors' therapeutic freedom, even if reimbursement may be excluded. However, this is not universal.

Authorization of the use of medicines to approved populations is not a valid reason for restricting their off-label use where this is warranted on clinical grounds. Other mechanisms exist, such as devolved budgets, physician education, 100% co-payment for prescribing outside of agreed indications and payback mechanisms. However, these can be labor-intensive and costly to administer (Adamski et al., 2010; Godman et al., 2012a, 2014; Ferrario and Kanavos, 2013; Malmstrom et al., 2013). EMA's report on Adaptive Licensing acknowledges the necessity of clear indications and we emphasize this point in this paper^4^. Vague or ambiguous definitions of indications are not helpful, e.g. when a new medicine is licensed for “patients for whom the [standard of care] is not appropriate.”

MAPPs imply that medicines approved in limited populations due to promising results of phase II trials are already being further researched whilst being prescribed in clinical care. It is important to repeat that this would further blur the boundary between research and routine use, and that patients would need to be informed about and accept this change (Husereau et al., 2014). It is also important to point out that clinical trials in populations for whom a medicine is already approved face certain obstacles. Such patients, unlike volunteers in randomised trials, may well not accept randomisation and blinding. Non-randomized, observational trials are more vulnerable to biases and they need to be designed even more meticulously than RCTs to avoid false results. Even then, the validity of observational trials is limited, compared to equally well designed, prospectively planned, randomized trials. We would expect to see more new medicines with unclear benefit-risk-assessments as MAPPs progresses. Currently, it is unclear how more data collected under adaptive pathways can compensate the inherent flaws with such approaches.

### Implications for the pricing of new medicines including negotiations

MAPPs aim to replace the so called “single magic moment” of approval by progressive management (Eichler et al., 2012; EMA^15^). From a payers' point of view, there may well be instances in which adaptive pathways are reasonable and necessary. However, there are concerns that high prices eventually granted for a targeted group of patients will be exploited when the numbers of indications are expanded, with considerable budgetary implications. One of the examples is ivacaftor, which already causes concerns to payers with its high requested price and comparatively limited patient benefits (Eichler et al., 2015; Godman et al., 2015). We are also already seeing a number of orphan medicines achieving “blockbuster” status as a result of such exploitation (Kesselheim et al., 2012). Differential pricing by indication is difficult for payers to cope with especially given limited routine availability of patient level data among countries and regions in Europe, MEAs difficult to administer and the appreciable use of external reference pricing among European countries (Adamski et al., 2010; Leopold et al., 2012; Ferrario and Kanavos, 2013).

In a recent publication, the authors expressed the hope that MAPPs might help to address the strain that high-priced medicines put on healthcare systems (Eichler et al., 2016). This can be disputed especially as the prices requested by pharmaceutical companies for their new medicines rarely mirror their development costs (Experts in Chronic Myeloid Leukemia, 2013). In addition, ever increasing prices are being charged for new medicines despite only a small minority of them considered as innovative by independent authorities (Kelly and Smith, 2014; Godman et al., 2015; Howard et al., 2015). The majority of new medicines are seen as similar or with marginal improvements over existing standards (Godman et al., 2015; Prescrire Editorial, 2016). This is especially the case for new cancer medicines despite similar development costs (Kantarjian et al., 2013). However, cancer medicines are not an isolated case. The prices of new medicines for hepatitis C have stimulated debates over reimbursed prices especially where there are appreciable differences between the costs of manufacture and requested prices (Phelan and Cook, 2014; de Bruijn et al., 2016), especially when the latter threaten health care budgets and their viability (Brennan and Shrank, 2014).

MEAs are an attempt to achieve risk sharing and flexible approaches to reimbursement and are perceived as an integral part of adaptive pathways that already involve payers. Currently, MEAs are not legally feasible in all EU Member States. Consequently, the need for this would result in an adjustment of national legislations. In addition, MEAs are predominantly in the control of pharmaceutical companies. As a result, they control which Member States are involved as well as the different conditions in Member States, depending on the Member State's power of negotiation (Ferrario and Kanavos, 2013). This leads to a distorted market and significantly different levels of access and affordability of new medicines for patients across Europe. From a public health or societal perspective, this is unacceptable. Apart from this, performance-based agreements under MEAs bear high transaction and administrative costs and are not easily implemented (Adamski et al., 2010; Ferrario and Kanavos, 2013, 2015). Adaptive pathways are not designed to lower these hurdles, which further underlines the belief among payers that they need to be low in number and limited to very special cases. Consequently, adaptive pathways cannot be the preferred approach for new medicines in the future.

### Shifting of research for new medicines to the post-authorisation phase—who should bear the costs?

There also are concerns regarding who should fund any additional costs to healthcare systems arising from higher prices for a new medicine versus existing standards during the conditional approval process as well as fund the data collection process. This is because increasing the amount of data generated post-licensing will result in a shift in costs. In the current environment, pre-marketing clinical trials are funded by pharmaceutical companies alone. If these costs are shifted to payers or the public, pharmaceutical companies need to come to an agreement with health authorities and health insurance companies on how the increased costs to healthcare systems will be compensated. Such costs include introducing comprehensive IT systems where currently it is problematic to collect comprehensive patient level data for the entire population.

### Enforcement—what if it doesn't work?

A central task in adaptive pathways will be to ensure that exit strategies are in place for new medicines not fulfilling expectations. No application should be complete without a full description of what will happen if patients do not respond to treatment. “No response” should be clearly defined in terms of the hoped-for outcomes and the consequences for all stakeholders. Revoking a marketing authorization has been difficult in the past and we do not expect it to be any easier in the future. This is no different for medicines approved with a conditional approval which do not manage to proceed to a standard approval. We have observed protests from doctors and patients likewise, independent of whether a medicine has been withdrawn or considered for withdrawal due to safety issues, lack of proven benefit or economic reasons (Raftery, 2010; Simoens et al., 2013). It has been indicated that rules for withdrawal could be part of MEAs (Eichler et al., 2015). In our experience, revoking a reimbursement decision can be even more difficult than revoking a marketing authorization; however the degree of difficulty depends on the local legal and cultural environment. Consequently for MAPPs to be workable, conditions for suspending marketing authorization of a new medicine should be included as part of any adaptive licensing process and decision. These should cover situations where there is a worsening of the risk-benefit-balance as more data becomes available and/ or where a company fails to generate the necessary data within defined time frames to continue to support requested prices. This process needs to be well-grounded in European law.

### Responsibility and liability for uncertainty and possible risk factors

In the context of adaptive pathways, a prohibition of product liability law suits during the initial marketing period has been proposed (Eichler et al., 2012). We see this proposal as unacceptable and not in line with European legislation. It also contradicts the official position of EMA that the current legislative framework is not to be changed. One cannot on the one hand open the way to earlier commercialisation and on the other hand transfer any risks arising from the new medicine to patients and the public domain. This liability prohibition exploits the special vulnerability of ill persons. This is even more questionable in the context of discussions about whether adaptively licensed products require even longer periods of intellectual property protection^6^. Earlier commercialisation, longer periods of exclusivity paired with reduced liability for damages gravely unbalance the concept of adaptive pathways.

## Ways forward/conclusions

Managing the entry of new medicines is undoubtedly necessary given the fact that a number of European countries are already struggling to fund new medicines at ever increasing prices (Malmstrom et al., 2013).

Whilst being open for constructive evaluation and dialogue on how the process of new drug development and their introduction can be improved, payers and their advisers have a number of concerns regarding current MAPPs to accelerate drug approval. This paper has aimed to describe these concerns and potential problems that arise from a payer perspective. Potential ways forward include developing new models to optimize the managed entry of new medicines as well as careful consideration which new medicines are applicable for the adaptive licensing process given existing procedures and concerns.

Suggested models for the introduction of new medicines build on payer concerns when dabigatran was being launched (Malmstrom et al., 2013; Permanand and Bak Pedersen, 2015). This started with extensive activities to combat pre-launch marketing in a number of countries. Payer concerns with dabigatran included the potential for excessive bleeding in elderly patients with atrial fibrillation with poor renal function, which were echoed in the deliberations between the marketing and medical departments of the company (Malmstrom et al., 2013; Cohen, 2014). An additional basis for future payer recommendations and involvement is also the constructive dialogue with all key stakeholder groups surrounding the development of pricing models for new medicines for orphan diseases—The Transparent Value Framework (Godman et al., 2015, European Commission^16^).

The introduction of new medicines into national health services is a trade-off between efficacy, safety, certainty and cost. Shifting the current balance to accelerate market access must consider not only the potential benefits, but also the potential harms to patients and society if a new medicine ultimately does not perform as originally expected.

Models that have been developed for managing the introduction of new medicines center on the three pillars of pre-, peri- and post-launch (please see Malmstrom et al. for further details). Pre-launch activities include horizon scanning and budgeting activities to identify new medicines that could have important implications for health services either in terms of potential costs, potential concerns as seen with dabigatran, or both. These can take place up to 3 years before potential EMA approval (Joppi et al., 2009; Wettermark et al., 2010; Godman et al., 2012b). Activities could also include educational activities as well as the development of quality indicators for use post launch. Peri-launch activities include all aspects of valuing and funding new medicines including reimbursement considerations as well as MEAs or risk-sharing arrangements (Adamski et al., 2010; Ferrario and Kanavos, 2013; Paris and Belloni, 2013). Post-launch activities include risk management plans (RMPs) as well as monitoring the effectiveness and safety of new medicines in routine clinical practice using electronic health records, registries or other approaches as well as monitoring the prescribing of medicines against agreed guidance and/ or quality indicators (Campbell et al., 2015; Godman et al., 2016).

The current tools and procedures for the management of new medicines would need to be further adapted and refined to meet the requirement of MAPPs. This involves a number of challenges including addressing the clarity of outcome parameters used and the time-frames connected with conditional approval and reimbursement. A redesign of the HTA process might be necessary to mirror the changes in the lifecycle of the products, particularly during the period of conditional approval and in their transition to a marketing authorization which is no longer conditional. Technologies for sophisticated interrogation of health authority databases with patient level data would be essential for the realization of concepts such as MAPPs. Currently, the use of these technologies is fraught with uncertainties and limitations.

EMA's report indicates that a great deal of work needs to be done to consistently ensure that any registries proposed for the collection of clinical outcomes across countries are fit for purpose^4^. Many post-authorization drug registries are fraught with potential biases. All too frequently data collection, data analysis and data interpretation are in the hands of, or heavily influenced by, the applicant. We have seen that this can lead to potential bias compared to independent registries (Marra et al., 2016). Consequently, requirements for balanced governance of registries should be in place.

From the perspective of academics researching the rational use of medicines, and from the perspective of payers and their advisers, concerns with the key issues described in this paper restricts the MAPPS pathway to medicines effectively addressing high unmet medical need, where high unmet need is narrowly defined.

It is not our intention as payers to create more barriers to the availability of new medicines where there is unmet medical need. However, payers need to make sure available funds provide comprehensive and equitable healthcare in all European countries and that one group of patients is not unfairly treated to the detriment of others. We hope this paper will stimulate important considerations and debates leading to developments to allay payer's concerns with adaptive pathways for new medicines.

## Author contributions

ME, ABu, MvdG, and AS developed the concept of the paper and produced the first draft. This was further developed by PVB, FA, MvdC, RS, RR and BG before all authors, i.e., ME, ABu, PVB, FA, ABy, TB, MvdC, ED, JF, KG, MvdG, JG, AH, JJ, RJ, OL, IL, AM, VM, LM, EM, EN, GS, CS, SS, RS, AS, RR, VV-P, CS and BG critiqued the final draft before submission.

## Funding

There was no funding body. However, the write-up work was in part supported by grants from the Karolinska Institutet, Sweden.

### Conflict of interest statement

The authors declare that the research was conducted in the absence of any commercial or financial relationships that could be construed as a potential conflict of interest.

## Footnotes

^1^Official Journal of the European Union. Council Conclusions on Common values and principles in European Union Health Systems (2006/C 146/01). Available at URL: http://eur-lex.europa.eu/legal-content/EN/TXT/PDF/?uri=CELEX:52006XG0622(01)&from=EN.

^2^European Council. Council conclusions on strengthening the balance in the pharmaceutical systems in the EU and its Member States. Available at URL: http://www.europa.eu/en/press/press-releases/2016/06/17-epsco-conclusions-balance-pharmaceutical-system/.

^3^EMA Adaptive Pathways Reply to Prof. Silvio Garattini, Peter C. Gøtzsche, Tom Jefferson, Joan-Ramon Laporte, Joel Lexchin, Donald W. Light, Martin McKee, Jean-Louis Montastruc, Sir Richard Thompson. Available at URL: http://www.ema.europa.eu/docs/en_GB/document_library/Other/2016/06/WC500208968.pdf.

^4^EMA Final report on the adaptive pathways pilot. Available at URL: http://www.ema.europa.eu/docs/en_GB/document_library/Report/2016/08/WC500211526.pdf.

^5^ADAPTSMART Accelerated Development of Appropriate Patient Therapies—a Sustainable, Multistakeholder Approach from Research to Treatment-outcomes. Available at URL: http://adaptsmart.eu/.

^6^ADAPT-SMART Project Overview. Available at URL: http://adaptsmart.eu/wp-content/uploads/2015/09/ProjectOverview-IMI2-ADAPTSMART.pdf.

^7^Joint briefing paper “Adaptive licensing” or “adaptive pathways”: Deregulation under the guise of earlier access. Available at URL: http://ec.europa.eu/health/files/committee/stamp/2015-10_stamp3/3c_prescrire_position_paper.pdf.

^8^EPHA Briefing: Will fast-tracking medicines improve affordability? Letter to Prof. Rasi and Dr Eichler. Available at URL: http://epha.org/wp-content/uploads/2016/05/Will-fast-tracking-for-new-medicines-improve-affordability-formatted-5.pdf.

^9^Adaptive pathways: EMA still leaves open questions unanswered. Current EMA report on the pilot project again documents perplexity regarding the use of “real world data”. Available at URL: https://www.iqwig.de/en/press/press-releases/press-releases/adaptive-pathways-ema-still-leaves-open-questions-unanswered.7492.html.

^10^First statistics on PRIME are released. Available at URL: http://www.ema.europa.eu/ema/index.jsp?curl=pages/news_and_events/news/2016/06/news_detail_002541.jsp&mid=WC0b01ac058004d5c1.

^11^Aussagekraft von Surrogatendpunkten in der Onkologie. 2011. Available at URL: https://www.iqwig.de/download/A10-05_Rapid_Report_Version_1-1_Surrogatendpunkte_in_der_Onkologie.pdf.

^12^EMA Annual Report 2015. Available at URL: http://www.ema.europa.eu/docs/en_GB/document_library/Annual_report/2016/05/WC500206482.pdf.

^13^“Adaptive licensing” or “adaptive pathways”: Deregulation under the guise of earlier access. Available at URL: http://english.prescrire.org/Docu/DOCSEUROPE/20151019_AdaptiveLicensing.pdf.

^14^Secret des affaires, Autorisations de mise sur le marché au rabais et opacité des essais cliniques. Available at URL: https://blogs.mediapart.fr/formindep/blog/010415/secret-des-affaires-autorisations-de-mise-sur-le-marche-au-rabais-et-opacite-des-essais-cliniques.

^15^Adaptive pathways to patients: report on the initial experience of the pilot project. Available at URL: http://www.ema.europa.eu/docs/en_GB/document_library/Report/2014/12/WC500179560.pdf.

^16^Process on Corporate Social Responsibility in the Field of Pharmaceuticals Platform on Access to Medicines in Europe Working Group on Mechanism of Coordinated Access to Orphan Medicinal Products (MoCA-OMP) - TRANSPARENT VALUE FRAMEWORK. Available at URL: http://ec.europa.eu/enterprise/sectors/healthcare/files/docs/orphans_conlusions_en.pdf2012.
